# eIF5A maintains intestinal epithelial homeostasis by sustaining intestinal stem cells

**DOI:** 10.1186/s13619-025-00243-z

**Published:** 2025-06-09

**Authors:** Leilei Li, Yanhui Xiao, Liansheng Liu, Qianying Zhang, Yong Zhang, Dahai Zhu, Ye-Guang Chen

**Affiliations:** 1 Guangzhou National Laboratory, Guangzhou, China; 2 The State Key Laboratory of Membrane Biology, Tsinghua-Peking Center for Life Sciences, School of Life Sciences, Tsinghua University, Beijing, China; 3 The MOE Basic Research and Innovation Center for the Targeted Therapeutics of Solid Tumors, School of Basic Medical Sciences, Institute of Biomedical Innovation, Jiangxi Medical College, Nanchang University, Nanchang, China; 4 Bioland Laboratory (Guangzhou Regenerative Medicine and Health Guangdong Laboratory), Guangzhou, Guangdong China; 5 State Key Laboratory for Complex, Severe, and Rare Diseases, Institute of Basic Medical Sciences, Chinese Academy of Medical Sciences and School of Basic Medicine, Peking Union Medical College, Beijing, China; 6 Institute of Basic Medicine, North Sichuan Medical College, Nanchong, Sichuan China

**Keywords:** Intestinal homeostasis, eIF5A, Intestinal stem cells, Mitochondrial translation

## Abstract

Intestinal homeostasis is sustained by self-renewal of intestinal stem cells (ISCs), which continuously divide and produce proliferative transit-amplifying (TA) and then progenitor cells. Eukaryotic translation initiation factor 5A (eIF5A), a conserved translation factor, involves in a variety of cellular processes, yet its role in intestinal homeostasis remains unclear. Here, we demonstrate that eIF5A is indispensable for maintaining intestinal epithelial homeostasis. Conditional knockout of *Eif5a* in the adult mouse intestinal epithelium leads to stem cell loss, suppressed cell proliferation, and increased apoptosis within the crypts, concurrent with shortened gut length, reduced mouse body weight and rapid animal mortality. Consistently, Eif5a deletion in intestinal organoids also exhibits resembling cellular phenotypes. Mass spectrometry analysis reveals a significant downregulation of mitochondrial proteins, particularly those involved in mitochondrial translation, upon eIF5A depletion. Analysis of a published single-cell RNA sequencing dataset shows that mitochondrial translation-related genes, including *Dars2*, are highly expressed in ISC, TA and progenitor cells. Furthermore, eIF5A-deficient organoids exhibit impaired mitochondrial function, characterized by reduced ATP levels and increased reactive oxygen species (ROS). These findings highlight a critical role for eIF5A in sustaining intestinal epithelial homeostasis by regulating mitochondrial translation, providing a new insight into the molecular mechanism underlying intestinal stem cell renewal and tissue maintenance.

**Supplementary Information:**

The online version contains supplementary material available at 10.1186/s13619-025-00243-z.

## Background

The small intestinal epithelium is structured into crypt-villus units, while the large intestine lacks the villus architecture. Remarkably, the intestinal epithelium in the homeostatic state exhibits a rapid renewal, with a turnover time of 3-5 days, making it the most rapidly self-renewing tissue in mammals (Barker 2014; Zhu et al. 2021). The constant renewal of the intestine epithelium is fueled by Lgr5^+^ intestinal stem cells (ISCs) situated at the base of the crypts (Beumer and Clevers 2021; Meng et al. 2022). These ISCs divide continuously, producing rapidly proliferating transit-amplifying (TA) cells and other proliferative progenitors (Haber et al. 2017; Liu et al. 2023; Wang et al. 2020; Wang et al. 2022). These daughter cells then migrate out of the crypt compartment and upward the villus as they differentiate into mature functional secretory cells (e.g., Paneth cells, goblet cells, enteroendocrine cells, and tuft cells) or absorptive enterocytes (Beumer and Clevers 2021; Clevers 2013).

Eukaryotic translation initiation factor 5A (eIF5A) is a highly conserved translation factor involved in regulating translational elongation and termination during protein synthesis (Schuller et al. 2017). It regulates a wide range of cellular processes, including cell cycle progression (Nakanishi et al. 2023), cell motility (Fujimura et al. 2015), autophagy (Zhang et al. 2019), and metabolism (Puleston et al. 2019). Notably, eIF5A has been shown to be essential for embryonic development and adult tissue homeostasis. For instance, eIF5A is required for mouse embryonic development (Nishimura et al. 2012), and it plays a crucial role in maintaining growth, viability, and neurodevelopment in the murine nervous system (Kar et al. 2021). Recent studies have further demonstrated that eIF5A is necessary for the activation of adult skeletal muscle stem cells in response to injury (Zhang et al. 2024). However, the role of eIF5A in intestinal epithelial homeostasis remains unclear.

In this study, we investigate the role of eIF5A in the maintenance of the intestinal epithelium. We demonstrate that eIF5A is essential for intestinal homeostasis, as its deletion leads to a loss of stem cells, suppression of cell proliferation, and induction of apoptosis in the intestinal epithelium. Mechanistically, eIF5A deficiency results in the downregulation of a variety of mitochondrial proteins, including a cohort of mitochondrial translation-related proteins. These results indicate that mitochondrial translation plays an important role in sustaining intestinal epithelium function.

## Results

### eIF5A is required for intestinal homeostatic maintenance

**Fig. 1:**
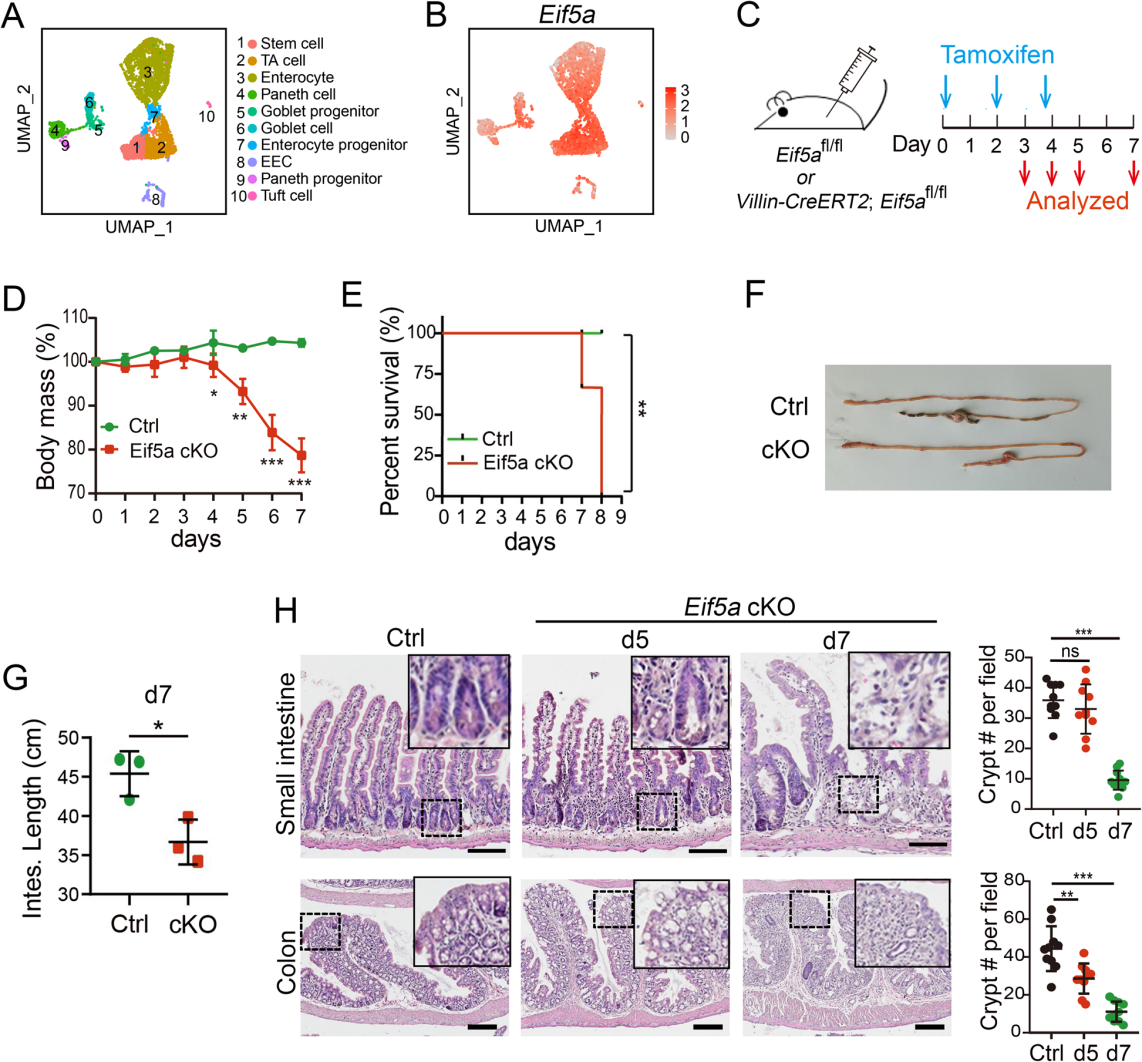
eIF5A is required for intestinal homeostatic maintenance. **A** Cell-type clusters of small intestinal epithelium were visualized by UMAP in scRNA-seq analysis of small intestinal epithelium. **B** The expression of *Eif5a* in different cell types were visualized by UMAP in scRNA-sequencing analysis of small intestinal epithelium. **C** Schematic of tamoxifen treatment regimen. Control (*Eif5a*^*fl/fl*^) and *Eif5a* cKO (*Villin-CreERT2; Eif5a*.^*fl/fl*^) mice were sacrificed and analyzed at indicated time points. **D** Body mass following TAM treatment. *n* = 4-5 mice/group. The body mass at individual day was compared. **E** Survival curve of mouse following TAM treatment. *n* = 6-7 mice/group. **F**, **G** Intestine image and intestinal length of Control and *Eif5a* cKO mice. *n* = 3 mice/group. **H** Left, H&E staining in small intestine and colon at the indicated time points. Right, Quantification of crypt number in small intestine (*n* = 10~11 random fields from 3 mice/group) and proximal colon (*n* = 10 random fields from 3 mice/group) at indicated time points. All the data represent mean ± SD. ***p < 0.001, **p < 0.01, *p < 0.05, ns = not significant, unpaired student-t test (**D**, **G**), Log-rank (Mantel-Cox) test (**E**), Mann-Whitney (two-tailed) U-test (**H**). Scale bars: upper, 100 μm; lower, 200 μm (**H**)

To uncover the role of eIF5A in intestinal homeostasis, we assessed its expression pattern in mouse small intestinal epithelium by analyzing a published single-cell RNA dataset (Liu et al. 2023), and found that *Eif5a* was ubiquitously expressed across all cell types of intestinal epithelium, with a relatively higher expression in Lgr5^+^ stem cells, TA cells, and progenitor cells (Fig. 1A-B and S1A-B). Consistently, immunofluorescence staining displayed a ubiquitous expression of eIF5A in the small intestinal and colonic epithelium (Fig. S1C-D).

To further investigate the role of eIF5A in intestinal epithelium, we generated inducible *Eif5a* conditional knockout (cKO) mice using the *Villin-CreERT2;Eif5a *^*fl/fl*^ mouse model (Fig. 1C). Immunofluorescence staining showed that eIF5A expression remained largely unchanged at 3 days post-tamoxifen treatment (dpt), showed a moderate decrease at 4 dpt, and was dramatically reduced by 5 dpt in tamoxifen-treated mice (Fig. S1C-1E). The deletion of *Eif5a* in intestinal epithelium caused a rapid-onset wasting phenotype, including body weight loss and pronounced morbidity, with affected mice exhibiting watery diarrhea, bloody stools, hypothermia, and a hunched posture, ultimately leading to lethality within 7~8 days (Fig. 1D-E). *Eif5a* deficiency resulted in marked intestinal shortening (Fig. 1F-G), severe disorganization of the intestinal epithelium structure, and loss of the crypt compartments in both the small intestine and colon at 7 dpt (Fig. 1H). Collectively, these results reveal an indispensable role of eIF5A in the maintenance of the intestinal epithelium.

### eIF5A deficiency impairs cell proliferation and eliminates stem cells in the intestinal epithelium

**Fig. 2:**
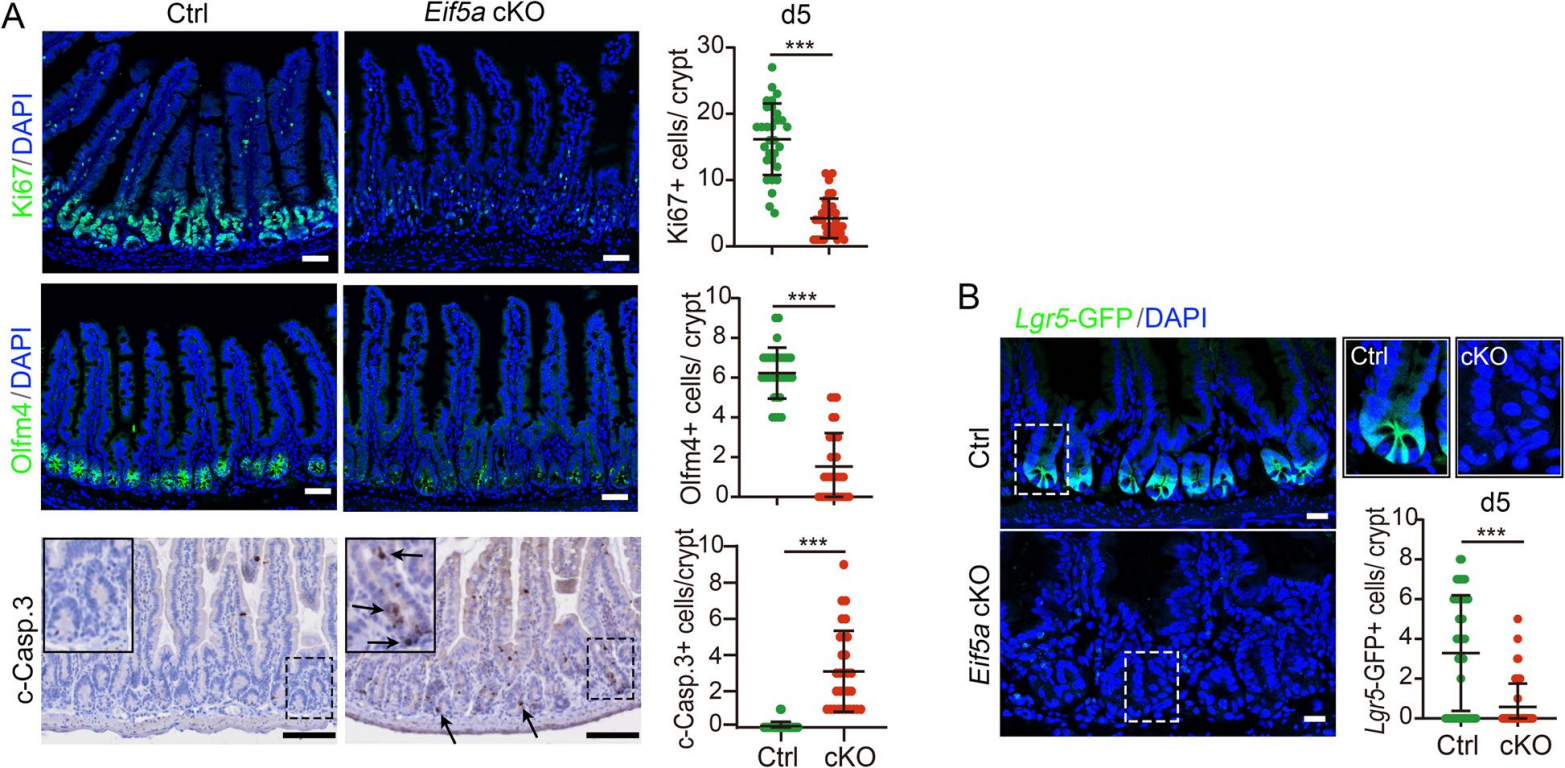
eIF5A deficiency impairs cell proliferation and stem cells in the intestinal epithelium. **A** Immunofluorescence or Immunohistochemistry staining images and quantification of Mki67^+^, Olfm4^+^ cells or c-Casp.3^+^ cells in small intestinal epithelium at 5 dpt. Upper, 30 random crypts from 3 mice/group were counted. Scale bars: 50 μm; Middle, 31-32 random crypts from 3 mice/group were counted, Scale bars: 50 μm; Lower, *n* = 30 random crypts from 3 mice/group were counted. Arrowhead indicates c-Casp.3 signals. Scale bars: 100 μm. **B** Immunofluorescence images and quantification of *Lgr5*-GFP^+^ cells in small intestinal epithelium from Control (*Lgr5-EGFP-IRES-CreERT2*; *Eif5a*^*fl/fl*^) treated with corn oil) and Eif5a cKO (*Villin-CreERT2*; *Lgr5-EGFP-IRES-CreERT2*; *Eif5a*^*fl/fl*^) treated with TAM) mice at 5 dpt. n = 52 random crypts from 2 mice/group. Scale bars: 50 μm. All the data represent mean ± SD. ***p < 0.001, *p < 0.05, Mann–Whitney (two-tailed) U-test. Nuclei were counter-stained with DAPI

Next, we investigated the impact of *Eif5a* deletion on cell composition of the intestinal epithelium. Immunofluorescence staining revealed a dramatic reduction in the number of proliferative cells (marked by Mki67) and stem cells (marked by Olfm4) in the *Eif5a*-cKO small intestine at 4 or 5 dpt (Fig. S2A, 2A). Similarly, a significant decrease in Mki67^+^ cells was also observed in the colon (Fig. S2B). Concurrently, apoptotic cells marked by cleaved caspase-3 showed a prominent increase in the crypts of both the small intestine and colon (Fig. 2A, S2B). In contrast, the number of lysozyme^+^ Paneth cells and Mucin2^+^ goblet cells remained unchanged. These cells are likely pre-existing post-mitotic cells, given that stem cells were suppressed in the Eif5a-deficient small intestine. Interestingly, Chga^+^ enteroendocrine cells exhibited a slight increase following eIF5A loss, indicating that eIF5A deficiency promotes their differentiation from the pre-existing progenitors (Fig. S2C).

To further assess the role of eIF5A in stem cell maintenance, we generated *Villin-CreERT2; Lgr5-EGFP-IRES-CreERT2; Eif5a *^*fl/fl*^ mice, in which ISCs were labeled by *Lgr5*-GFP. Consistently, the number of *Lgr5*-GFP^+^ ISCs was markedly reduced in both small intestinal and colonic epithelium upon *Eif5a* KO (Fig. 2B, S2D). Taken together, these findings demonstrate that eIF5A plays an indispensable role in promoting cell proliferation, survival, and the maintenance of intestinal stem cells.

### eIF5A is essential for cell proliferation and survival in intestinal organoids

**Fig. 3:**
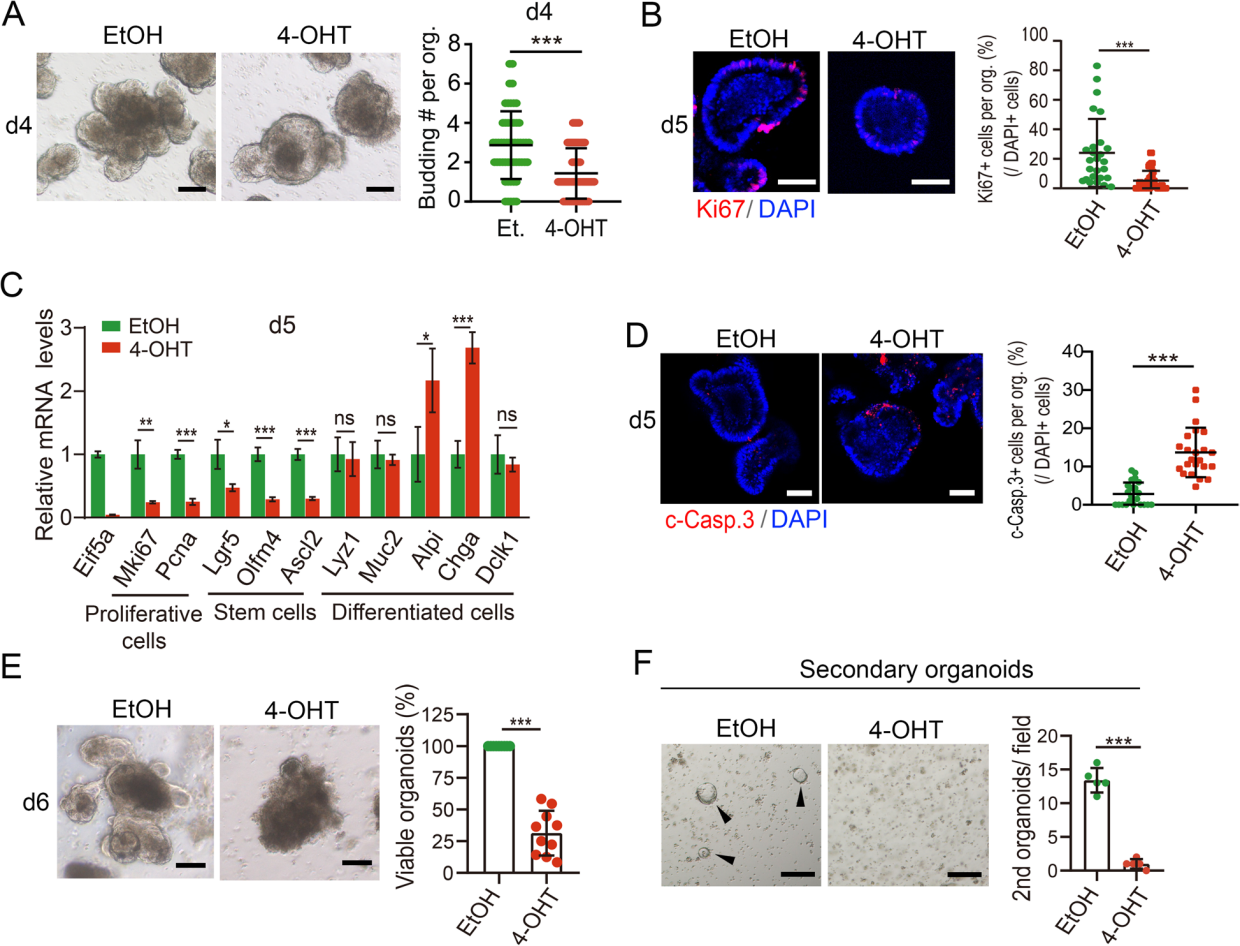
eIF5A is necessary for cell proliferation and survival in intestinal organoids. **A** Images and quantification of budding in small intestinal organoids (*Villin-CreERT2*; *Eif5a*^*fl/fl*^) 5 days following EtOH (vehicle) or 4-OHT treatment. Arrowhead shows bud. n = 53 organoids from 4-5 replicates per group. Data were from one of three independent experiments. **B** Immunofluorescence staining of Mki67 in the organoids 5 days following indicated treatments. Percentage of Mki67^+^ cells (relative to DAPI^+^ cells) were quantified. n = 49-55 organoids from 7-8 random fields/group. Data were from one of two independent experiments. **C** The relative expression levels of indicated marker genes were assessed by RT-qPCR in the organoids at day 5 following EtOH or 4-OHT treatment. Data were from one of three independent experiments. **D** Immunofluorescence staining of c-Casp.3 in the organoids 5 days following EtOH or 4-OHT treatment. Percentage of c-Casp.3^+^ cells (relative to DAPI^+^ cells) were quantified. n = 23 organoids from 9-12 random fields/group. Data were from one of two independent experiments. **E** Images and quantification of viable organoids (relative to total organoids/field) 6 days following EtOH or 4-OHT treatment. 9-10 fields/group. Data were from one of three independent experiments. Viable organoids were identified by the presence of a distinct and intact epithelial cell border. Organoids with a dark appearance, broken and indistinguishable cell borders throughout the entire body were considered non-viable. **F** Secondary organoids formed from passaged fragments of organoids treated with EtOH or 4-OHT. n = 5 random fields from three replicate wells/group. Organoids were passaged 3 days post treatment and the secondary organoids formed were imaged and counted 1-2 days after passaging. Data were from one of three independent experiments. Arrowhead indicates the organoids. All the data represent mean ± SD. ***p < 0.001, **p < 0.01, *p < 0.05, Mann-Whitney (two-tailed) U-test (**A**, **B**, **D**, **E**), unpaired student-t test (**C**). Scale bars: 50 μm (**B**, **D**), 100 μm (**A**, **E**), 20 μm (**F**). Nuclei were counter-stained with DAPI

Next, we further investigated the role of eIF5A in the intestinal epithelium using organoids. Small intestinal organoids were established from the crypts isolated from *Villin-CreERT2; Eif5a*^*fl/fl*^ mice, and *Eif5a* deletion in organoids was induced by 4-hydroxytamoxifen (4-OHT) treatment (Fig. S3A). Following *Eif5a* knockout, the organoid bud formation and Mki67^+^ cells were significantly reduced (Fig. 3A-B); Concurrently, the mRNA levels of proliferative and stem cell marker genes were downregulated, whereas differentiated cell markers remained largely unchanged, with upregulation of the enterocyte marker *Alpi* and the enteroendocrine cell marker *Chga* (Fig. 3C), implying a potential role of eIF5A in modulating enterocyte and enteroendocrine cell differentiation. Notably, *Eif5a*-deficient organoids exhibited a dramatic increase in cleaved caspapse-3^+^ (c-Casp.3^+^) apoptotic cells (Fig. 3D), and cell death (Fig. 3E, S3B). These data further demonstrate the critical role of eIF5A in promoting cell proliferation and survival and preventing cell death, thereby maintaining intestinal stem cells.

To assess whether eIF5A is required for secondary organoid formation, which reflects the renewal capacity of organoid-forming stem cells, we passaged *Eif5a*-deficient organoids. The organoids showed a nearly complete loss of secondary organoid generation (Fig. 3F), indicating that eIF5A is essential for the regenerative potential of intestinal stem cells.

### eIF5A deficiency leads to the downregulation of mitochondrial translation-related proteins

**Fig. 4:**
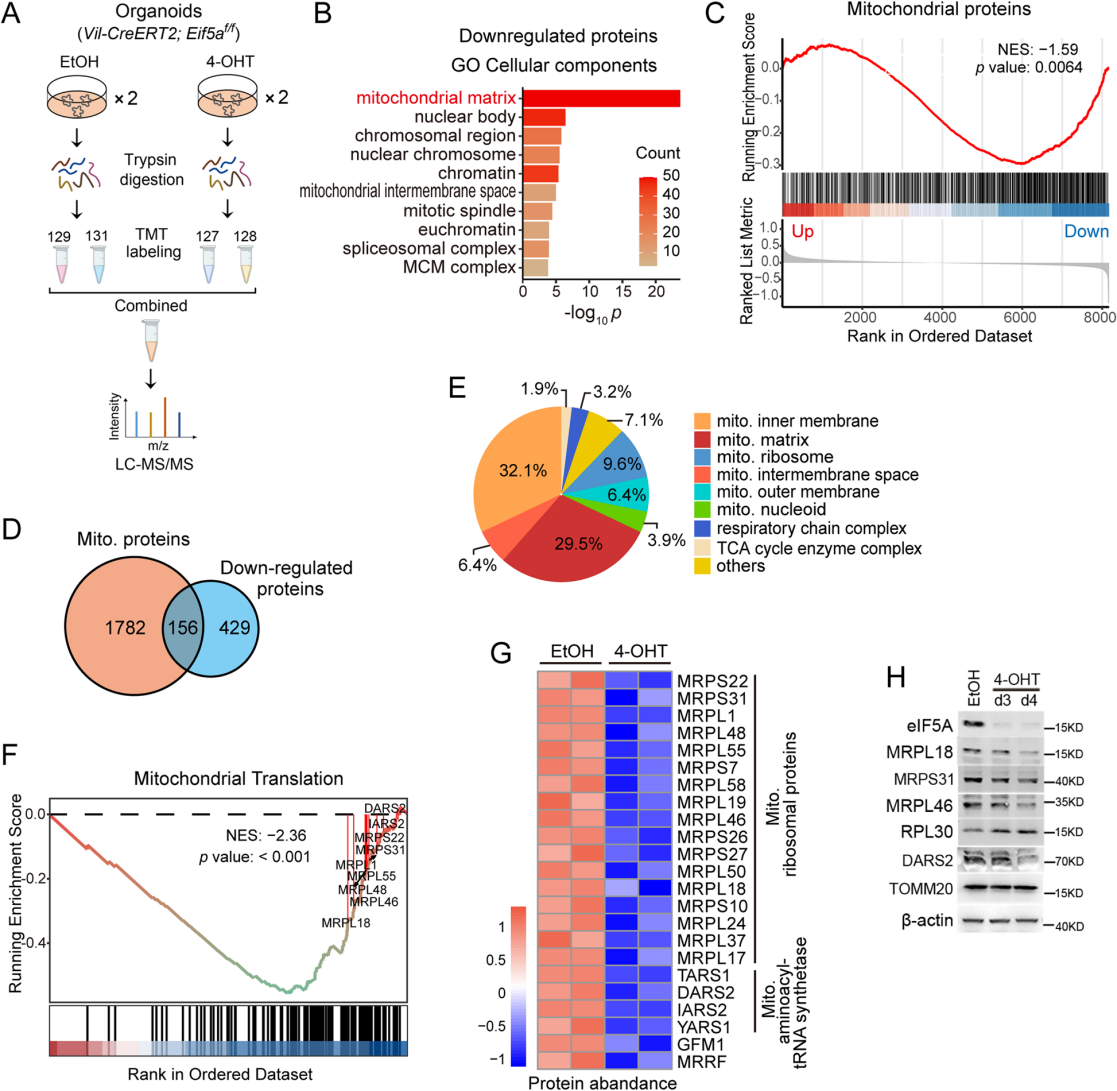
*Eif5a* depletion leads to the downregulation of mitochondrial translation-related proteins. **A** Outline of the proteome strategy. Small intestinal organoids (*Villin-creERT2*; *Eif5a*^*fl/fl*^) treated with either EtOH or 4-OHT were collected on day 3 post-treatment for TMT-based LC-MS/MS analysis. Two biological replicates/group. **B** Gene ontology (GO) cellular components of Eif5a loss-downregulated proteins were analyzed using Metascape web server. **C** An enrichment of mitochondrial proteins was shown by gene set enrichment analysis (GSEA). The mitochondrion protein list was from mouse gene ontology term (GO:0005739). **D** Venn diagram shows the overlapped ones between 1938 mitochondrion proteins and 585 downregulated proteins affected by Eif5a. **E** Pie charts show the distribution and percent of the downregulated mitochondrial proteins affected by Eif5a in different mitochondrion compartments. **F** An enrichment of mitochondrial translation was shown by GSEA. **G** The heatmap displays mitochondrial translation-related proteins that were downregulated. Scaled by Z-score. **H** The indicated protein expressions were examined by immunoblotting in small intestinal organoids (*Villin*-creERT2; Eif5a^*fl/fl*^) treated with either EtOH or 4-OHT at indicated time points

To investigate the underlying mechanism of eIF5A’s role in the intestinal epithelium, we performed tandem mass tag (TMT)-based liquid chromatography-tandem mass spectrometry (LC-MS/MS) to identify downstream proteins regulated by eIF5A (Fig. 4A). We identified 585 downregulated proteins and 328 upregulated proteins in intestinal organoids following *Eif5a* knockout (Table S1). Gene Ontology (GO) analysis showed that the downregulated proteins were mainly enriched in a variety of metabolic processes (Fig. S4A), while the upregulated proteins had an enrichment in the protein involved in positive regulation of apoptotic process (Fig. S4B), consistent with the observed increase in c-Casp.3^+^ apoptotic cells in intestinal tissues. Interestingly, GO cellular component analysis highlighted a striking localization of the downregulated proteins in the mitochondrial matrix (Fig. 4B-C). Indeed, Gene Set Enrichment Analysis (GSEA) confirmed a significant downregulation of mitochondrial proteins upon *Eif5a* loss. Among these, 156 mitochondrial proteins were downregulated (Fig. 4D), with a broad distribution across mitochondrial compartments (Fig. 4E). Intriguingly, a subset of mitochondrial translation-related proteins, including mitochondrial ribosomal proteins (MRPs) and several mitochondrial aminoacyl-tRNA synthetases, were significantly downregulated (Fig. 4F-G). In contrast, a number of cytoplasmic translation-related proteins, including cytoplasmic ribosomal proteins (RPs), were upregulated (Fig. S4C-D), likely as a compensatory response to the suppression of mitochondrial translation following *Eif5a* knockout. To validate these findings, we selected several MRPs (MRPL18, MRPS31, MRPL46), a cytoplasmic ribosomal protein (RPL30), and a mitochondrial aminoacyl-tRNA synthetase (DARS2) for analysis by immunoblotting (Fig. 4H). The results confirmed their downregulation or upregulation in *Eif5a*-depleted organoids. However, the expression of another mitochondrial protein, TOMM20 (translocase of outer mitochondrial membrane 20), remained unchanged (Fig. 4H). Collectively, these data reveal that eIF5A plays a pivotal role in regulating the expression of mitochondrial proteins, including a subset of mitochondrial translation-related proteins.

**Fig. 5:**
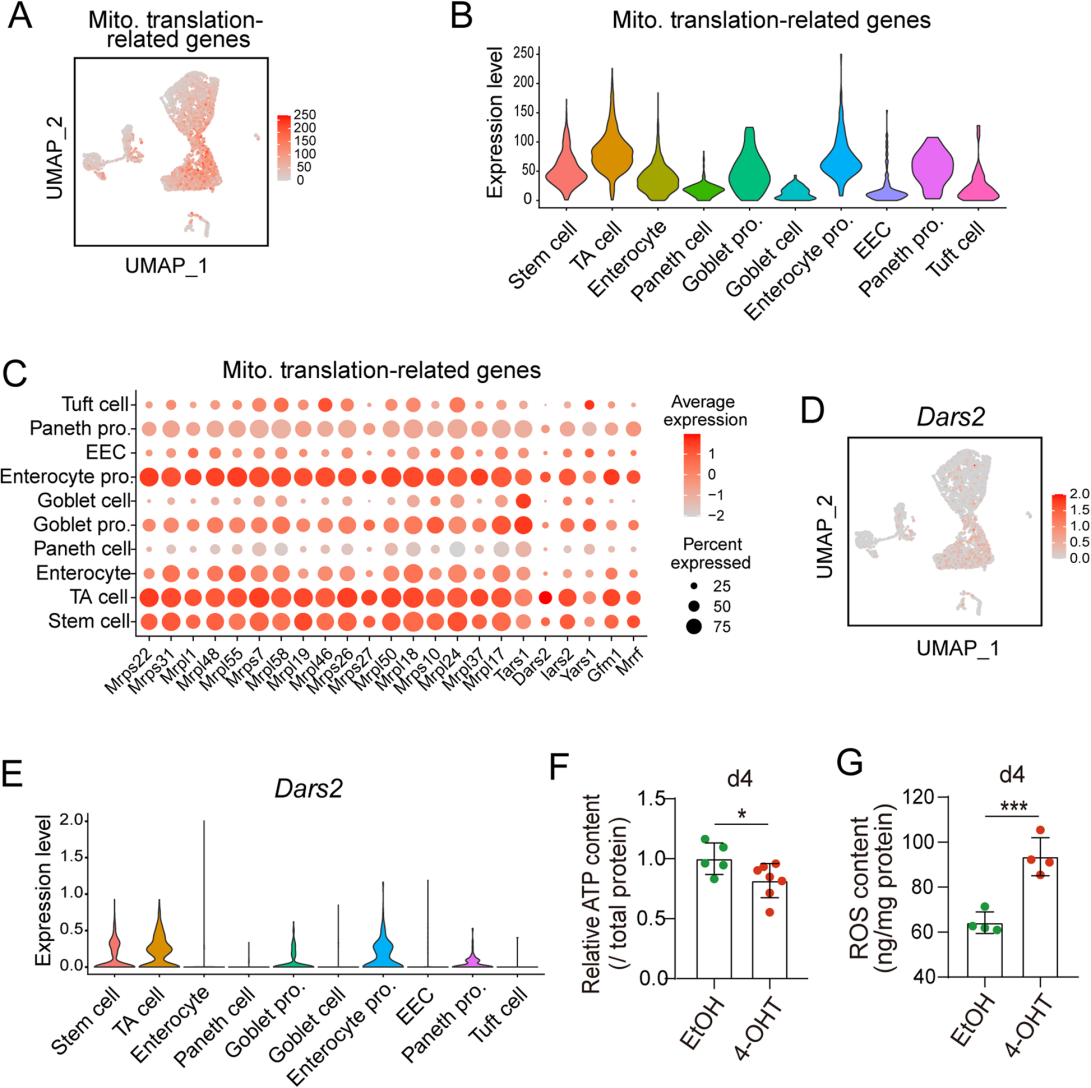
*Eif5a* depletion leads to the downregulation of mitochondrial translation-related proteins. **A** Expression of the metagene expression per single cell of the mitochondrial translation-related genes were visualized by UMAP in scRNA-sequencing analysis of small intestinal epithelium. **B** Violin plots showing the metagene expression levels per single cell of the mitochondrial translation-related genes in different cell types. Paneth pro., Paneth progenitor; EEC, Enteroendocrine cell; Enterocyte pro., Enterocyte progenitor; Goblet pro., Goblet progenitor. **C** Dot plot of mitochondrial translation-related genes in different cell types in scRNA-sequencing analysis of small intestinal epithelium. Dot color saturation indicates the average expression level (scaled by Z-score), and the dot size indicates the percentage of cells expressing the gene. **D** Expression of *Dars2* were visualized by UMAP in scRNA-sequencing analysis of small intestinal epithelium. **E** Violin plots show *Dars2* expression in different cell types. **F**, **G** Cellular ATP or ROS content was measured in small intestinal organoids (*Villin-CreERT2*; *Eif5a *^*fl/fl*^) 4 days following EtOH/4-OHT treatment. ATP (5-7 biological replicates/group) or ROS (4 biological replicates/group) levels were normalized to the total protein amount. The data represent mean ± SD from one of two independent experiments. ***p < 0.001, *p < 0.05, unpaired student-t test

Next, we assessed the expression pattern of these mitochondrial translation-related genes using single-cell RNA sequencing data from small intestinal epithelium. These genes were broadly expressed across all cell types, with relatively higher expression in the stem cells, TA cells, and progenitor cells (Fig. 5A-C), suggesting their potential involvement in sustaining cell proliferation and stem cell maintenance in the intestinal epithelium. Specifically, *Dars2*, which has been reported to play an essential role in maintaining stem cells and supporting cell proliferation during mouse intestinal epithelial homeostasis (Moschandrea et al. 2024), was highly expressed in stem cells, TA cells, and progenitor cells, but showed low expression in post-mitotic differentiated cells (Fig. 5D-E). Finally, we assessed mitochondrial function in *Eif5a*-deficient organoids. As expected, cellular ATP levels were significantly reduced, while reactive oxygen species (ROS) levels were markedly increased upon *Eif5a* loss (Fig. 5F-G), indicating impaired mitochondrial function. Taken together, these findings suggest a potential role of eIF5A-regulated mitochondrial translation in sustaining mitochondria function and the maintenance of intestinal homeostasis.

## Discussion

eIF5A is a highly conserved translation factor that functions in the translation elongation and termination. It undergoes hypusine modification at lysine 50, a post-translational modification crucial for its in vivo activity (Guo and Zhou 2024). The hypusination of eIF5A is a two-step enzymatic process catalyzed by two key enzymes: deoxyhypusine synthase (DHPS) and deoxyhypusine hydroxylase (DOHH) (Nakanishi and Cleveland 2024). A previous study has shown that specific depletion of *Dhps*, which abolishes eIF5A hypusination, in the mouse intestinal epithelium using a Villin-Cre genetic model leads to gut inflammation and epithelial distortion, characterized by enlarged crypt regions (Gobert et al. 2023). In contrast, we demonstrate that conditional knockout of *Eif5a* in the adult mouse intestinal epithelium using a Villin-CreERT2 model results in the loss of stem cells and the crypt compartment. One possible explanation for the phenotypic differences between these models may be due to the timing and context of gene deletion: deletion occurs from the developing embryonic stage (Villin-Cre) or specifically during the adult homeostatic state (Villin-CreERT2). This distinction suggests that developmental context and potential compensatory mechanisms may critically influence the observed phenotypes. Additionally, another possible explanation for the discrepancy between the phenotypes of *Eif5a* and *Dhps* knockout is that *Dhps* knockout might not completely abolish the function of eIF5A.

eIF5A hypusination-dependent synthesis of mitochondrial proteins has been described in the liver (Zhou et al. 2022), brain (Liang et al. 2021; Schroeder et al. 2021), and immune cells (Puleston et al. 2019). It has been reported that hypusinated eIF5A promotes translation elongation by alleviating ribosome stalling at amino acid motifs that cause structural constraints, including consecutive polyproline motifs, conserved non-polyproline tripeptide sequences (Doerfel et al. 2013; Gutierrez et al. 2013; Pavlov et al. 2009; Schuller et al. 2017; Ude et al. 2013), and mitochondrial targeting sequences (MTSs) present in mitochondrial proteins (Zhou et al. 2022). Consistent with these findings, our study demonstrates that eIF5A regulates the protein levels of a subset of mitochondrial proteins, particularly those involved in mitochondrial translation, in the intestinal epithelium. The presence of MTSs in these proteins may, in part, confer a dependency on eIF5A during their synthesis. However, other potential mechanisms by which eIF5A regulates mitochondrial proteins in the intestine cannot be ruled out.

Translational control of protein synthesis is critical for stem cell functions and disease development (Gay et al. 2022; Robichaud et al. 2019; Saba et al. 2021). Selective translation is increasingly recognized as a major determinant of functional specificity for distinct protein subsets in a cell type-specific manner. eIF5A has been well-documented for its regulatory role in the selective translation of protein subsets in different cellular context (Lubas et al. 2018; Puleston et al. 2019; Zhang et al. 2024). Here, the eIF5A-mediated selective translation of a subset of mitochondrial proteins in the intestinal epithelium provides crucial insights into the translational control of intestinal stem cell maintenance and gut homeostasis.

Mitochondria play a vital role in intestinal stem cell maintenance and epithelial homeostasis (Rath et al. 2018). Here, we established that a cohort of mitochondrial translation-related proteins are enriched in stem cells, transit-amplifying (TA) cells, and progenitor cells. We propose that eIF5A-mediated regulation of these proteins, such as DARS2 that has a role in intestinal stem cell maintenance (Moschandrea et al. 2024), is crucial for maintaining intestinal epithelial homeostasis.

## Methods

### Mice

*Villin-creERT2* mice were kindly provided by Dr. Sylvie Robine (Institut Curie, France). *Eif5a*^*fl/fl*^ mice with *loxP* sites flanking exon 3 of the eukaryotic translation initiation factor 5 A (*Eif5a*) gene were generated in Dr. Dahai Zhu’s laboratory (Zhang et al. 2024). Both mice strains were on a C57BL/6 genetic background. The *Villin-creERT2*; *Eif5a*^*fl/fl*^ mice were generated by crossing the above two lines together. *Lgr5-EGFP-IRES-CreERT2* mice were obtained from the Jackson Laboratory, and crossed with *Villin-creERT2*; *Eif5a*^*fl/fl*^ animals to generate *Villin-creERT2*; *Lgr5-EGFP-IRES-CreERT2*; *Eif5a *^*fl/fl*^ mice. All mouse strains were bred and housed at the animal facility with specific pathogen free (SPF) conditions. All animal studies were performed in accordance with the relevant guidelines, under the approval of the Institutional Animal Care and Use Committee of Guangzhou National Laboratory (approval number: GZLAB-AUCP- 2023-01-A01).

For conditional knockout of Eif5a in intestinal epithelium, 8–10 week-old *Villin*-*creERT2*; *Eif5a*^*fl/fl*^ mice were intraperitoneally injected with Tamoxifen (TAM) (Sigma, stock 20 mg/ml in corn oil) at a dose of 100 mg/kg body mass every 24 h for three consecutive days. For the assessment of *Lgr5*-GFP^+^ cells in intestinal epithelium, *Villin-creERT2*; *Lgr5-EGFP-IRES-CreERT2*; *Eif5a*^*fl/fl*^ mice were treated with tamoxifen using the above regime, and the littermate *Lgr5-EGFP-IRES-CreERT2*; *Eif5a*^*fl/fl*^ mice parallelly injected with corn oil were used as the control. The following PCR primers (5’-3’) were used for the genotyping of WT and *Eif5a*^*fl/fl*^ mice:

Forward primer, CTTCTCCTGGGTTATCGTCTTCA; Reverse primer, CTCACAACACTTGCCCAGCAG.

### Crypt isolation and organoid culture

Mouse small intestinal crypts were isolated and cultured as previously described (Li et al. 2025). Briefly, the small intestines were isolated and cut longitudinally, then washed with cold PBS for three times. The villi were completely scraped away and small pieces (1 cm) of intestines were incubated in 2 mM EDTA in PBS for 30 min at 4 °C. These pieces were then vigorously suspended in cold PBS and the mixture was subsequently passed through 70 mm cell strainer (BD Biosciences) for purification. The crypt fractions were enriched through centrifugation at a speed of 400-500 g for 3 min, then embedded in Matrigel (Corning) and seeded on a 24-well plate in ENR culture medium (advanced DMEM/F12 supplemented with penicillin/streptomycin, GlutaMax, N2, B27, and N-acetylcysteine, containing 50 ng/mL EGF, 100 ng/mL Noggin, and 500 ng/mL R-spondin1) and refreshed every 2-3 days. For ex vivo* Eif5a* depletion, vehicle (ethanol, 1:1,000) or 1 μM 4-OHT (Sigma) was added directly into the medium for 24 h.

The secondary organoids formation experiments were conducted as previously described (Stine et al. 2019). Briefly, small intestinal organoids (*Villin-CreERT2*; *Eif5a*^*fl/fl*^) were collected 3 days following EtOH or 4-OHT treatment. The organoids were trypsinized for two minutes in TrypLE Express (Invitrogen) at 37 °C to generate fragments, which were plated into three replicate wells per group. The number of organoids that formed were imaged and counted from random fields in each well 1-2 days after passaging.

All quantifications were conducted based on the captured images from random fields. Viable organoids were defined by the presence of a distinct and intact epithelial cell border. Organoids exhibiting dark appearance, broken, and undistinguishable cell border in the total body were considered as dead ones. ‘‘Non-budding’’ or spheroid organoids were characterized by a round or oval shape without any epithelial protrusions. “Budding” referred to the emergence of an epithelial protrusion representing a crypt from the main spheroid body.

### Immunofluorescence, Immunohistochemistry and H&E staining

Immunofluorescence, immunohistochemistry, and histological staining were conducted as previously described (Qi et al. 2017). or according to the standard procedures. H&E staining was performed according to the manufacturer instruction (Beyotime Biotechnology, #C0205S). The primary antibodies used were as following: rabbit anti-eIF5 A (1:100, Proteintech, #11,309-1-AP), mouse anti-E-cadherin (1:200, BD Transduction Laboratories, #610,182), rabbit anti-Ki67 (1:200, Abcam, ab15580), rabbit anti-Olfm4 (1:500, CST, Cat#19,141), rabbit anti-Cleaved Caspase 3 (1:100, CST, #9664L), rabbit anti- Lysozyme (1:500, Abcam, ab108508), rabbit anti-Chga (1:500, Abcam, ab15160), rabbit anti-Mucin2 (1:1000, Abcam, ab272692); Secondary antibodies were goat anti-mouse IgG, Alexa Fluor 488 (1:1000, ThermoFisher, A11029) and goat anti-rabbit IgG, Alexa Fluor 594 (1:1000, ThermoFisher, A11037) and DAPI (1:1000, Roche). Intestinal tissue sections were visualized with a confocal microscope (Zeiss, LSM980).

For organoid staining, the procedure was conducted according to the previous describes (Stine et al. 2019). Briefly, organoids were collected and fixed with 4% PFA. Primary antibodies were incubated overnight, organoids were washed and secondary antibody was incubated for 1 h at room temperature. Finally, organoids were mounted onto a cell culture dish (Nest, 801,001) for imaging using a Leica LSM980 confocal microscope.

### RNA extraction and qRT-PCR

Small intestinal organoids were collected. Total RNA was extracted and then reverse transcribed into cDNA using HiScript III RT SuperMix for qPCR (+ gDNA wiper) (Vazyme, R323-01). Real-time PCR was performed on a CFX96 Real-Time PCR Detection System (Bio-rad) using SYBR green fluorescent dye (Vazyme, Q711-02). Fold changes were calculated by using the delta delta CT method, with A*ctb* as a reference gene for normalization. Primers were listed in Table S2.

### Immunoblotting

The organoids were collected and lysed in RIPA buffer (Genstar, E122-01) with protease inhibitors (Roche). And total protein was quantified using a BCA protein assay kit (Beyotime, P0012S), 20-40 μg of total protein was separated with 10% SDS PAGE gel under denaturing conditions and was transferred to nitrocellulose membranes (PALL, #66485). The membranes were blocked and incubated with the primary antibodies and subsequent secondary anti-rabbit or anti-mouse conjugated antibodies. Anti-Eif5a were used as listed above, and others were as following: rabbit anti-MRPL18 (Proteintech, # 15178-1-AP), rabbit anti-MRPS31 (Proteintech, # 16288-1-AP), rabbit anti-MRPL46 (Proteintech, # 16611-1-AP), rabbit anti-RPL30 (Proteintech, # 17403-1-AP), rabbit anti-DARS2 (Proteintech, # 13807-1-AP), rabbit anti-TOMM20 (Proteintech, #11802-1-AP), rabbit anti-β-actin (Beijing Zhongshan Golden Bridge Biotechnology Co. Ltd, #TA-09).

### TMT-based LC–MS/MS analysis

Small intestinal organoids (*Villin*-creERT2; *Eif5a*^*fl/fl*^) were treated with either EtOH or 4-OHT (Two biological replicates/group) for 24 h and collected on day 3 post-treatment. Organoid pellets were lysed with PBS buffer containing 8 M urea (pH 8.0), 1 mM PMSF, and 1 mM protease inhibitor cocktail. A protein solution containing 100 µg of protein was prepared for enzymatic digestion. The proteins were reduced with 10 mM DTT and alkylated with 40 mM Iodoacetamide, and then digested with sequencing grade modified trypsin (Promega, Fitchburg, WI) at 37 °C overnight. The digested peptides were desalted using Sep-Pak columns (Waters, Milford, MA, USA). Peptides from different samples were labeled with tandem mass tag (TMT) reagents (Thermo Fisher Scientific, Pierce Biotechnology, Rockford, IL, USA). TMT127, TMT128, TMT129, and TMT131 were used for labeling KO and control samples. The TMT-labeled peptides were mixed and further desalted using a Sep-Pak column. The peptides were fractionated using a UPLC3000 system (Dionex, Sunnyvale, CA, USA) equipped with an XBridge BEH300 C18 column (Waters, Milford, MA, USA). Forty-eight fractions were collected, dried using a speedvac, combined into 12 fractions, and re-dissolved in 0.1% formic acid (FA).

For LC-MS/MS analysis, the TMT-labeled peptides were separated using a 120-min gradient elution at a flow rate of 0.30 µL/min with a Thermo-Dionex Ultimate 3000 HPLC system, which was directly interfaced with a Thermo Scientific Orbitrap Exploris 480 mass spectrometer. The analytical column was a custom-made fused silica capillary column (75 µm ID, 350 mm length) packed with C-18 resin (1.9 µm, Dr. Maisch GmbH). The mobile phase consisted of 0.1% formic acid, and mobile phase B consisted of 80% acetonitrile and 0.1% formic acid. The Orbitrap Exploris 480 mass spectrometer was operated in data-dependent acquisition mode using Xcalibur software. This involved a single full-scan mass spectrum in the Orbitrap (350-1600 m/z, 60,000 resolution) followed by 2 s of data-dependent MS/MS scans in an Ion Routing Multipole at 32 normalized collision energy (HCD).

The MS/MS spectra from each LC-MS/MS run were searched against the human.fasta database using an in-house Proteome Discoverer (Version PD2.5, Thermo Fisher Scientific, USA). The search criteria included fully tryptic specificity, allowance for up to two missed cleavages, and setting carbamidomethylation (C) and TMT sixplex (K and N-terminal) as fixed modifications. Oxidation (M) was set as a variable modification. Precursor ion mass tolerances were set at 20 ppm for all MS acquired in an Orbitrap mass analyzer, and the fragment ion mass tolerance was set at 0.02 Da for all MS2 spectra. Confidence levels were set to achieve a 1% FDR (high confidence).

### Measurement of ATP or ROS content in organoids

Small intestinal organoids derived from *Villin-CreERT2*; *Eif5a*^*fl/fl*^ mice were treated with either ethanol (EtOH, vehicle control) or 4-hydroxytamoxifen (4-OHT) for 24 h and collected on day 4 post-treatment. Organoid pellets were lysed, and ATP content was measured using a commercial ATP assay kit (Vazyme Biotech, Cat# DD1102) according to the manufacturer’s instructions. A standard ATP solution (Beyotime Biotechnology, Cat# D7378) was used to generate a standard curve for quantification. Meanwhile, a portion of the cell lysate was used to measure total protein amount using a BCA protein assay kit (Solarbio, Cat# PC0020). ATP contents were normalized to the total protein amount.

Cellular ROS levels were measured using a ROS ELISA kit (Shanghai Enzyme-linked Biotechnology, Cat# YJ009876) following the manufacturer’s protocol. Then, ROS levels were normalized to the total protein content measured using the same BCA protein assay kit.

### Bioinformatics analysis

GO analysis were conducted using the Metascape web server (Zhou et al. 2019). GSEA was performed using an online tool (https://www.bioinformatics.com.cn). The figures in the single-cell RNA-sequencing analysis were plotted with R software (v4.3.2).

### Statistics

All experiments presented in this study were independently repeated at least two times, unless explicitly indicated in the main text or figure legends. Exact numbers of mice, fields, and organoids are shown in the figure legends. Crypt, organoid, or cell number difference between two groups were compared using Mann-Whitney (two-tailed) U-test. Difference between two groups in other assays were compared using unpaired Student’s t-test. All data shown in graphs represent mean ± SD, *p < 0.05, **p < 0.01, ***p < 0.001. The statistical analysis was performed with Graph-Pad Prism (version 8.0).

## Supplementary Information

Supplementary Material 1 Fig. S1. eIF5A is required for intestinal homeostatic maintenance. Fig. S2. eIF5A deficiency impairs cell proliferation and stem cells in the intestinal epithelium. Fig. S3. eIF5A is necessary for cell proliferation and survival in intestinal organoids. Fig. S4. *Eif5a* depletion leads to the downregulation of mitochondrial translation-related proteins.

Supplementary Material 2 Table S1. The proteomes in intestinal organoids following *Eif5a* knockout.

Supplementary Material 3 Table S2. The primers used in this paper.

## Data Availability

All the single-cell level analyses were performed using a publicly available single-cell RNA-sequencing dataset from wild type mouse small intestinal epithelium (accession number: GSE186913). All data generated or analyzed during this study are included in this published article and its supplementary information files.

## Abbreviations

ISCs: Intestinal stem cells
TA: Transit-amplifying
eIF5A: Eukaryotic translation initiation factor 5A
TMT: Tandem mass tag
LC-MS/MS: Liquid chromatography-tandem mass spectrometry
GO: Gene Ontology
GSEA: Gene Set Enrichment Analysis
MRPs: Mitochondrial ribosomal proteins
RPs: Ribosomal proteins
ROS: Reactive oxygen species
