# Supplementary material for: eIF5A maintains intestinal epithelial homeostasis by sustaining intestinal stem cells
Source: Cell Regen. 2025 Jun 9;14:23. doi: 10.1186/s13619-025-00243-z (PMC12149079; doi:10.1186/s13619-025-00243-z)
Supplement: Supplementary file 1 — Supplementary Material 1 Fig. S1. eIF5A is required for intestinal homeostatic maintenance. Fig. S2. eIF5A deficiency impairs cell proliferation and stem cells in the intestinal epithelium. Fig. S3. eIF5A is necessary for cell proliferation and survival in intestinal organoids. Fig. S4. Eif5a depletion leads to the downregulation of mitochondrial translation-related proteins. [file 13619_2025_243_MOESM1_ESM.docx]

**Supplementary Figures**

**eIF5A maintains intestinal epithelial homeostasis by sustaining intestinal stem cells**

Leilei Li^1^, Yanhui Xiao^1^, Liansheng Liu^1^, Qianying Zhang^4, 5, 6^, Yong Zhang^4, 5^,

Dahai Zhu^4, 5^ and Ye-Guang Chen^1, 2, 3 *^

Figures S1 to S4


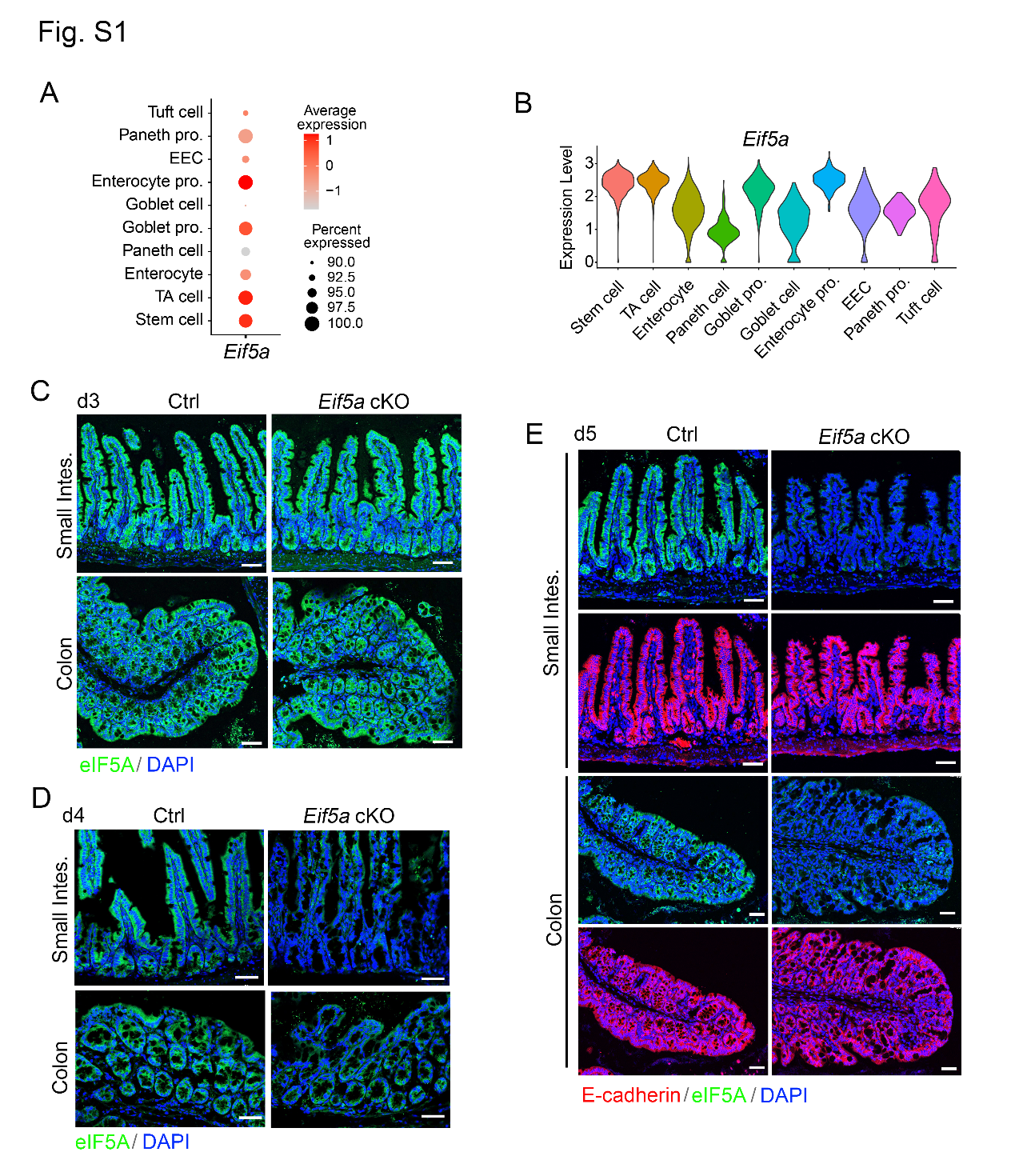


**Figure S1.** **eIF5A is required for intestinal homeostatic maintenance.**

(A) Dot plot of *Eif5a* expression in different small intestinal cell types. Dot color saturation indicates the average expression level (scaled by Z-score), and the dot size indicates the percentage of cells expressing the gene. Paneth pro., Paneth progenitor; EEC, Enteroendocrine cell; Enterocyte pro., Enterocyte progenitor; Goblet pro., Goblet progenitor.

(B) Violin plots showing the expression levels of *Eif5a* per single cell in different cell types.

(C, D, E) Immunofluorescence staining of eIF5A in small intestine and colon at 3, 4 or 5 dpt.

Scale bars: 50 μm. Nuclei were counter-stained with DAPI.


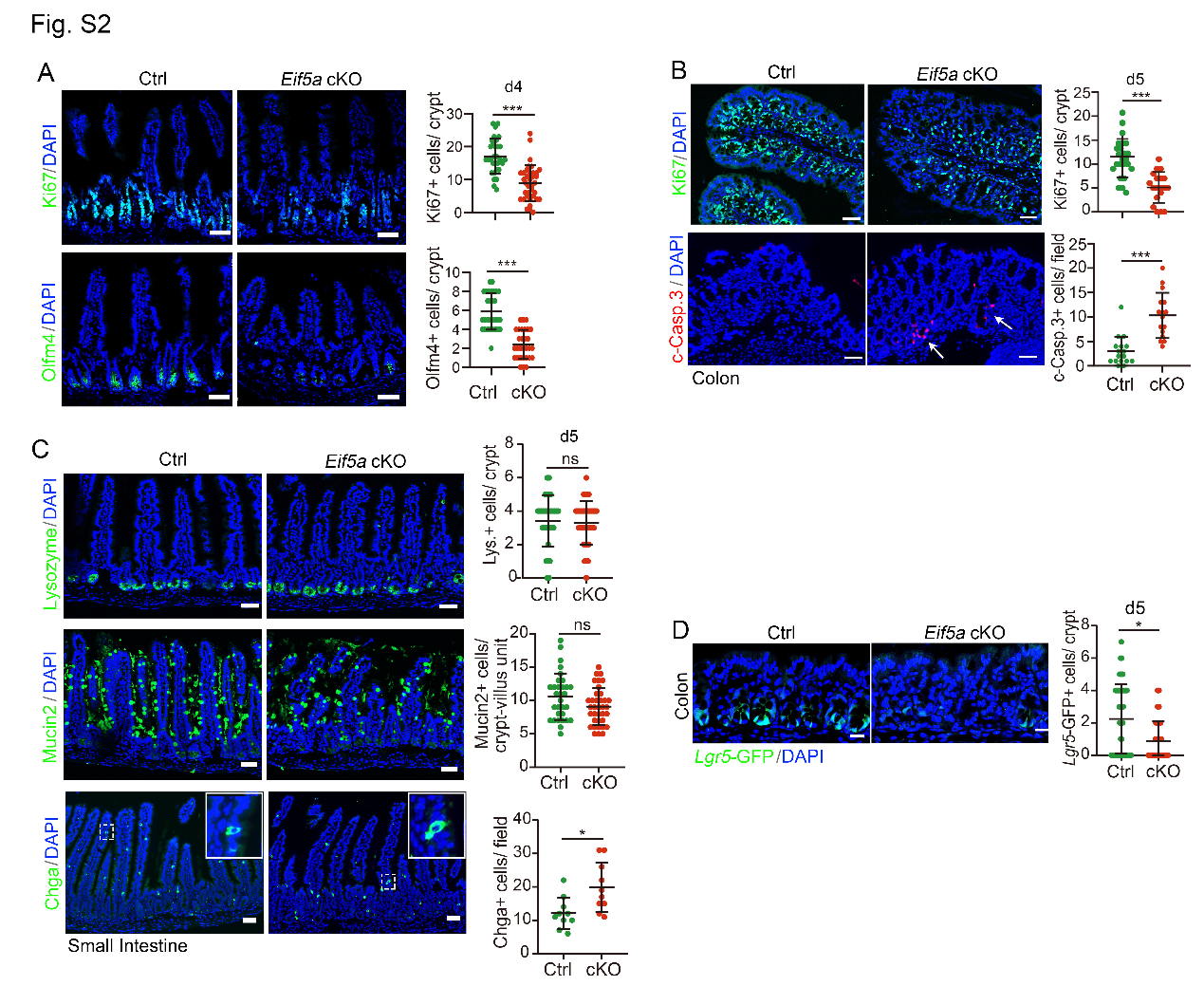


**Figure S2. eIF5A deficiency impairs cell proliferation and stem cells in the intestinal epithelium**

(A) Immunofluorescence images and quantification of Mki67^+^ and Olfm4^+^ cells in small intestine at 4 dpt. Upper, n = 31-36 random crypts from 3 mice/ group.Lower. n = 30 random crypts from 3 mice/ group.

(B) Immunofluorescence images and quantification of Mki67^+^ and c-Casp.3^+^ cells cells in proximal colon at 5 dpt. Upper, n = 19-24 random crypts from 3 mice/ group.Lower. n = 16-17 random fields from 3 mice/ group.

(C) Immunofluorescence images or quantification of Lysozyme^+^ cells (n = 31-36 crypts from 3 mice/group), Mucin2^+^ cells (n = 31-36 crypt-villus units from 3 mice/group), and Chga^+^ cells (n = 10 fields from 3 mice/group) in small intestine at 5 dpt.

(D) Immunofluorescence images and quantification of *Lgr5*-GFP^+^ cells in proximal colon from Control (*Lgr5-EGFP-IRES-CreERT2*; *Eif5a^fl/fl^*) or Eif5a cKO (*Villin-CreERT2*; *Lgr5-EGFP-IRES-CreERT2*; *Eif5a^fl/fl^*) mice at 5 dpt. n = 50-52 random crypts from 2 mice/ group.

All the data represent mean ± SD, ***p < 0.001, **p < 0.01, *p < 0.05, ns= not significant, Mann-Whitney (two-tailed) U-test. Scale bars: 50 μm (A-D). Nuclei were counter-stained with DAPI.


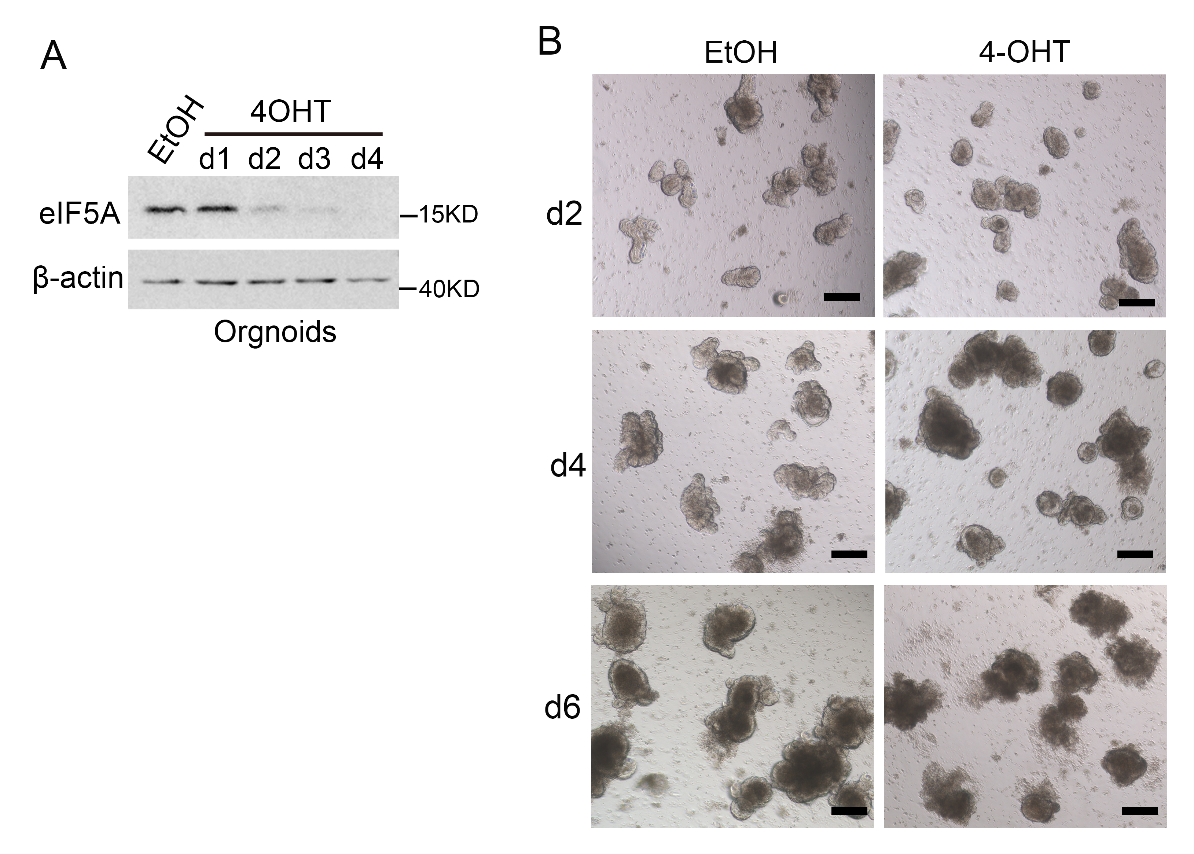


**Figure S3. eIF5A is necessary for cell proliferation and survival in intestinal organoids.**

(A) eIF5A protein levels were examined by immunoblotting in organoids (*Villin-CreERT2*; *Eif5a^fl/fl^*) at indicated time points post EtOH or 4-OHT treatment.

(B) Images of *Villin-CreERT2*; *Eif5a^fl/fl^* organoids at indicated time points following EtOH or 4-OHT treatment. Scale bars: 200 μm.


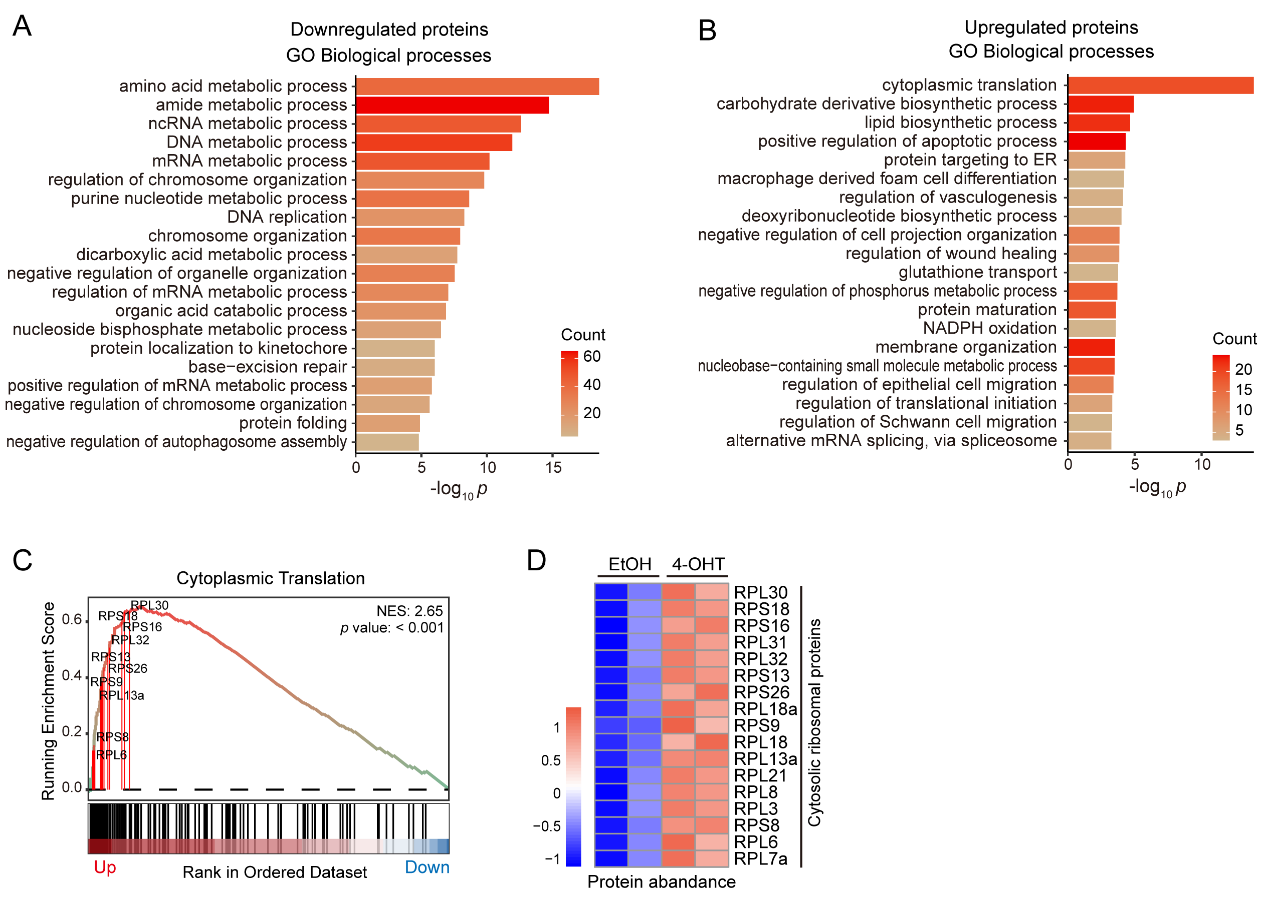


**Figure S4. *Eif5a* depletion leads to the downregulation of mitochondrial translation-related proteins.**

(A, B) GO enrichment analysis was performed on the downregulated (585 genes) or upregulated genes (328 genes) (p≤0.05) identified by TMT-based LC-MS/MS. Metascape was used for the analysis.

(C) An enrichment of cytoplasmic translation was shown by gene set enrichment analysis (GSEA).

(D) The heatmap displays cytoplasmic ribosomal-related proteins that were upregulated.
